# Impact of admission serum total cholesterol level on in-hospital mortality in patients with acute aortic dissection

**DOI:** 10.12669/pjms.324.10124

**Published:** 2016

**Authors:** Xintian Liu, Xi Su, Hesong Zeng

**Affiliations:** 1 Xintian Liu, Department of Cardiology, Tongji Hospital, Tongji Medical College, Huazhong University of Science and Technology, Wuhan, China; 2 Xi Su, Department of Cardiology, Wuhan Asia Heart Hospital, Wuhan, China; 3 Hesong Zeng, Department of Cardiology, Tongji Hospital, Tongji Medical College, Huazhong University of Science and Technology, Wuhan, China

**Keywords:** Acute aortic dissection, In-hospital mortality, Propensity score matching, Total cholesterol

## Abstract

**Objective:**

To find out the association between serum total cholesterol (TC) on admission and in-hospital mortality in patients with acute aortic dissection (AAD).

**Methods:**

From January 2007 to January 2014, we enrolled 1492 consecutive AAD patients with serum TC measured immediately on admission. Baseline characteristics and in-hospital mortality were compared between the patients with serum TC above and below the median (4.00 mmol/L). Propensity score matching (PSM) was used to account for known confounders in the study. Cox proportional hazard model was performed to calculate the hazard ratio (HR) and 95% confidence interval (CI) for admission serum TC levels.

**Results:**

With the use of PSM, 521 matched pairs of patients with AAD were yielded in this analysis due to their similar propensity scores. Patients with admission serum TC < 4.00 mmol/L, as compared with those with admission serum TC ≥ 4.00 mmol/L, had higher in-hospital mortality (11.7% vs. 5.8%; HR, 2.06; 95% CI, 1.33-3.19, P = 0.001). Stratified analysis according to Stanford classification showed that the inverse association between admission serum TC and in-hospital mortality was observed in patients with Type-A AAD (24.0% vs. 11.3%; HR, 2.18; 95% CI, 1.33 - 3.57, P = 0.002) but not in those with Type-B AAD (3.8% vs. 2.2%; HR, 1.71; 95% CI, 0.67 - 4.34, P = 0.261).

**Conclusions:**

Lower serum TC level on admission was strongly associated with higher in-hospital mortality in patients with Type-A AAD.

## INTRODUCTION

Acute aortic dissection (AAD) remains a catastrophic cardiovascular disease.^1^ Overall about 20% of patients with AAD and aneurysm died before reaching hospital, 30% or so during hospital admission, and a further 20% over the next ten years.^2^ Hence, predictive markers to identify AAD patients at increased risk of death are valuable for risk stratification and guiding treatment.

Total cholesterol (TC) is a well-known risk factor for cardiovascular diseases and a significant predictor of adverse outcome.^3^,^4^ Lipid-lowering therapy reduces mortality and improves clinical prognosis in patients with various cardiovascular diseases.^5^ However, the prognostic role of serum TC levels on admission hasn’t been clarified in patients with AAD. Besides, recent guidelines have insufficient evidence to recommend measuring serum TC levels and using lipid-lowering therapy for patients with AAD.^6^ The aim of the current study was to estimate the prognostic value of admission serum TC levels in patients with AAD.

## METHODS

### Study population

A total of 1492 consecutive patients with AAD were admitted from January 2007 to January 2014 in two hospitals (Tongji Hospital and Wuhan Asia Heart Hospital) in China. Patients with history of Marfan syndrome, systemic inflammatory disease, cancer, recent chest trauma, recent intervention and recent surgery were excluded. Moreover, patients with recent lipid-lowering therapy and incomplete data on any variables required for this study were also excluded. The study was approved by the Ethics Committees of the Tongji hospital and Wuhan Asia Heart Hospital. Both Ethics Committees specially approved that the requirement of informed consent was waived because data were going to be analyzed anonymously.

### Definitions

The diagnosis of aortic dissection was based on the results of history, transthoracic echocardiography and contrast-enhanced CT. Patients with AAD is defined as patients admitting to hospital within 14 days after the onset of AAD symptoms. According to Stanford classification, the extent of AAD is categorized into Type-A AAD and Type-B AAD (whether involving the ascending aorta). Smoking and alcohol drinking were divided as never and ever. Hypertension was defined by a clinic record of systolic blood pressure ≥ 140 mmHg and diastolic blood pressure ≥ 90 mmHg. Diabetes mellitus was defined as self-reported physician’s diagnosis of diabetes, fasting glucose level ≥ 7.8 mmol/L, or glucose level ≥ 11.1 mmol/L at two hour after oral glucose challenge. The primary endpoint of the study was all-cause in-hospital mortality.

### Laboratory assessments

Blood samples were drawn immediately on admission to the hospital. Serum TC and other biochemical variables (blood urea nitrogen, creatinine, uric acid, alanine aminotransferase, blood glucose, high-sensitive C-reactive protein and D-dimer) and hematological variables (leukocyte, hemoglobin, platelet) were measured by standard laboratory procedures on a Modular DP (Roche Diagnostics) and LH750 (Beckman Coulter), respectively.

### Statistical analysis

Patients with AAD were divided into two groups according to the median value of TC (4.00 mmol/L) in our study. Baseline characteristics between the low and high TC group in patients with AAD were compared with Student’s t test for continuous variables and with χ^2^ test for dichotomous variables. Natural logarithmic transformation was used for positively skewed variables whenever appropriate. Kaplan-Meier method and Cox proportional hazard model were used to estimate the prognostic value of serum TC on in-hospital mortality in patients with AAD.

Given the differences in baseline characteristics between the low and high serum TC level group in patients with AAD, propensity score matching (PSM) was used to identify a cohort of participants with similar baseline to reduce potential confounding in this observational study.^7^ Propensity scores were calculated with the use of a logistic regression model, with serum TC levels (above and below 4.00 mmol/L) as dependent variables, and with all baseline variables listed in Table-I as independent variables. We conducted PSM by using a 1:1 nearest neighbor matching protocol without replacement, with a caliper width equal to 20% of the standard deviation of the logarithm of the propensity score. Meanwhile the low and high TC group were exactly 1:1 matched on Stanford classification with the use of PSM module in SPSS software. Model fit was evaluated by Hosmer-Lemeshow goodness of fit test and the C-statistic test. After PSM, the baseline characteristics were compared with a paired t test for continuous variables and the McNemar test for categorical variables. Post match balance was assessed by standardized difference, which less than 10% for a given covariate suggests adequate balance. A two-tailed *P* value less than 0.05 was considered statistically significant. All statistical analyses were performed with SPSS V.19.

**Table-I:** Baseline characteristics according to admission serum TC categories in patients with AAD before and after PSM.

	Before PSM			After PSM		

	TC < 4.00 mmol/L	TC ≥ 4.00 mmol/L	P	TC < 4.00 mmol/L	TC ≥ 4.00 mmol/L	P
Number of patients	748	744		521	521	
Age, years	53.3 (11.6)	53.1 (11.1)	0.770	53.3 (11.6)	52.9 (10.8)	0.577
Sex			0.100			0.594
Men, %	80.7	77.3		78.1	79.7	
Women, %	19.3	22.7		21.9	20.3	
Smoking, %	59.4	59.3	0.974	56.6	56.8	1.000
Alcohol drinking, %	44.1	48.7	0.079	46.1	46.4	0.950
Hypertension, %	82.8	87.9	0.005	86.0	86.4	0.931
Diabetes, %	3.6	4.8	0.238	3.8	4.6	0.652
Stanford classification			<0.001			1.000
Type-A, %	45.9	33.2		39.2	39.2	
Type-B, %	54.1	66.8		60.8	60.8	
Time since AAD onset to admission			<0.001			0.792
≤ 24 h, %	59.4	49.1		44.1	43.2	
> 24 h, %	40.6	50.9		55.9	56.8	
Heart rate, beats/min	80.6 (16.8)	81.1 (15.0)	0.525	80.3 (16.1)	81.2 (14.3)	0.319
SBP, mmHg	142.7 (29.0)	150.2 (27.7)	<0.001	146.6 (28.9)	148.8 (27.0)	0.191
DBP, mmHg	80.0 (19.1)	83.8 (18.3)	<0.001	81.9 (18.8)	83.3 (17.8)	0.214
Leukocyte, 10^9^/L	11.4 (3.9)	11.8 (3.7)	0.049	11.4 (3.7)	11.5 (3.5)	0.680
Hemoglobin, g/L	124.8 (19.4)	133.0 (17.7)	<0.001	129.0 (17.3)	130.1 (17.5)	0.217
Platelet, 10^9^/L	172.2 (76.1)	178.5 (69.3)	0.093	176.4 (73.9)	177.9 (73.2)	0.736
BUN, mmol/L	7.7 (4.5)	6.8 (3.1)	<0.001	7.1 (3.8)	7.0 (3.4)	0.471
Creatinine*, umol/L	4.5 (0.5)	4.4 (0.4)	<0.001	4.5 (0.4)	4.4 (0.4)	0.370
Urea acid, umol/L	341.8 (130.5)	347.6 (113.8)	0.366	336.3 (117.0)	337.3 (112.3)	0.881
ALT*, IU/L	3.2 (1.0)	3.1 (0.7)	0.003	3.0 (0.8)	3.1 (0.7)	0.569
RGB, mmol/L	7.7 (3.5)	7.7 (2.5)	0.951	7.6 (2.8)	7.6 (2.2)	0.829
Hs-CRP*, mg/L	3.2 (1.4)	2.8 (1.3)	<0.001	3.0 (1.4)	3.1 (1.2)	0.773
D-dimer*, ug/ml	1.5 (0.7)	1.4 (0.7)	0.005	1.4 (0.7)	1.4 (0.7)	0.234
Treatment			0.002			0.723
Medicine, %	44.2	48.7		45.1	43.2	
Intervention, %	39.0	30.6		34.4	36.3	
Surgery, %	16.8	20.7		20.5	20.5	

Data are mean (SD) or %, unless otherwise noted.

* Natural logarithmic transformation. AAD, acute aortic dissection; TC, total cholesterol; SBP, systolic blood pressure; DBP, diastolic blood pressure; BUN, blood urea nitrogen; ALT, alanine aminotransferase; Hs-CRP, high-sensitive C-reactive protein; RBG, random blood glucose; PSM, propensity score matching.

## RESULTS

The baseline characteristics in AAD patients according to admission serum TC categories before and after PSM are presented in Table-I. Before PSM, there were major differences between the low TC group (< 4.00 mmol/L) and the high TC group (≥ 4.00 mmol/L) in several of the baseline variables. With the use of PSM, 521 matched pairs of AAD patients (Hosmer-Lemeshow goodness of fit test *P* = 0.48; C-statistic = 0.71) were yielded in the study. The low and high TC groups were exactly 1:1 matched on Stanford classification. There were no longer any significant differences between the low and high serum TC group for any covariates after PSM. The highest standardized differences less than 10% for all baseline variables indicated only minor differences between the two groups.

**Fig.1:**
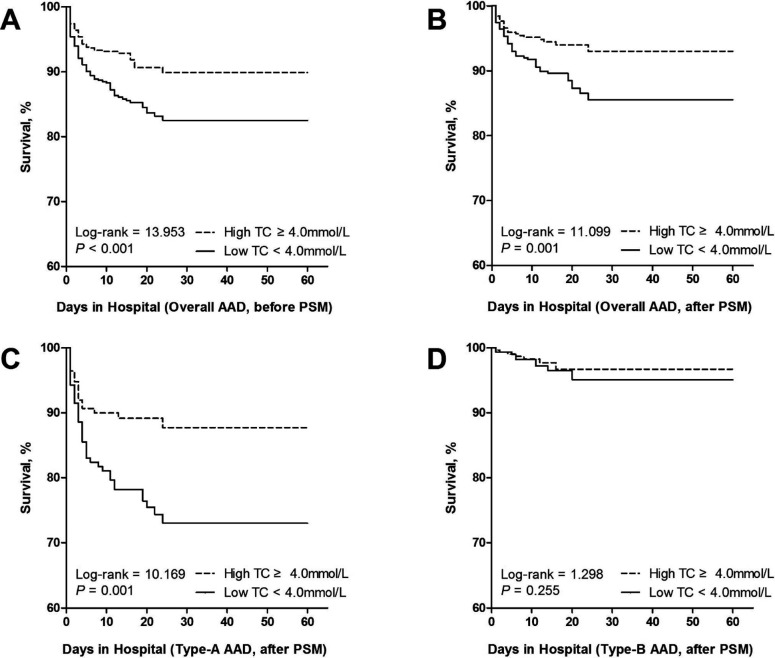
Kaplan-Meier curve showing cumulative survival rate in hospital according to admission serum TC categories in AAD patients before PSM (A), in AAD patients after PSM (B), in Type-A AAD patients after PSM (C) and in Type-B AAD patients after PSM (D). AAD, acute aortic dissection; TC, total cholesterol; PSM, propensity score matching.

In the 1492 AAD patients, mean serum TC was 4.08 ± 0.93 mmol/L, with TC ranging from 0.63 to 10.28 mmol/L. After PSM, mean serum TC were 3.44 ± 0.41 mmol/L (ranging from 1.52 to 3.99 mmol/L) and 4.71 ± 0.61 mmol/L (ranging from 4.00 to 7.29 mmol/L), in the low and high TC group, respectively. The median (interquartile range) of corresponding hospital stays were 12 (4-20) days and 12 (6-18) days, respectively. In-hospital survival analysis showed that the low TC group had significant higher all-cause in-hospital mortality than did the high TC group before PSM (15.4% *vs*. 9.0%, *P* < 0.001; Fig.1A). This conclusion also applied to the data after PSM (*P* = 0.001; Fig.1B). Stratified analysis according to Stanford classification showed that the low TC group was associated with an increased in-hospital mortality in patients with Type-A AAD (*P* = 0.001; Fig.1C), but not in those with Type-B AAD (*P* = 0.255; Fig.1D). The Cox proportional hazards regression analysis demonstrated that the HR (95% CI) of in-hospital mortality in Type-A AAD patients with admission serum TC < 4.00 mmol/L was 2.18 (1.33 - 3.57) after PSM (*P* = 0.002, Table-II).

**Table-II:** Risk of in-hospital mortality by admission serum TC categories in AAD patients after PSM.

	Number of death	Event rate, %	HR (95% CI)	P value
Overall AAD				
TC < 4.00 mmol/L (n=521)	61	11.7	2.06 (1.33-3.19)	0.001
TC ≥ 4.00 mmol/L (n=521)*	30	5.8	1.00	
Type-A AAD				
TC < 4.00 mmol/L (n=204)	49	24.0	2.18 (1.33-3.57)	0.002
TC ≥ 4.00 mmol/L (n=204)*	23	11.3	1.00	
Type-B AAD				
TC < 4.00 mmol/L (n=317)	12	3.8	1.71 (0.67-4.34)	0.261
TC ≥ 4.00 mmol/L (n=317)*	7	2.2	1.00	

* Conference group. AAD, acute aortic dissection; TC, total cholesterol; PSM, propensity score matching; HR, hazard ratio; CI, confidence interval.

## DISCUSSION

In this propensity analysis, we found that a lower serum TC level on admission was significantly associated with higher in-hospital mortality in patients with AAD. Further stratified analysis according to Stanford classification showed that the inverse relationship between admission serum TC and in-hospital mortality was observed in patients with Type-A AAD but not in those with Type-B AAD.

Although high TC is a well-known risk factor for cardiovascular diseases, low TC was found to be related with worse prognosis and higher mortality in cardiovascular diseases, such as coronary heart disease^8^ and heart failure.^9^ This inverse epidemiology is known as the “cholesterol paradox”.^10^ There was a similar conclusion in the present study. The findings in our study suggest that the hazardous effect of low TC appeared to be prominent in patients with Type-A AAD rather than in patients with Type-B AAD. Moreover, instead of developing a false sense of security in Type-A AAD patients with low TC, these patients may in fact need more intensive care and therapy.

The mechanism underlying this inverse association between TC level and adverse prognosis is presently unclear. One explanation might be based on the concept that TC is an indicator of nutritional status. High TC might reflect better nutritional status, which is likely associated with better tolerance of acute medical stress. In contrast, lower TC might reflect reduced food intake and intestinal absorption due to bowel edema and may be a result of increased metabolic stress.^11^ This may partly explain why low TC is related with the poor outcome of AAD. Furthermore, it should be noted that low TC is associated with in-hospital mortality in Type-A but not in Type-B AAD. This might be because Type-A AAD is much more dangerous than Type-B AAD. In the study, in-hospital mortality in Type-A AAD was 23.9% (141/590), while in Type-B AAD was 4.5% (41/902). Higher mortality reflects higher stress, catabolism and nutritional consumption, resulting in TC reduction.^12^ In addition, other nutritional parameters, such as albumin and triglyceride, are also linked with mortality.^13^-^15^

### Limitations of the study

Firstly, although PSM was used to account for known potential confounders in the study, the relationship between serum TC levels and in-hospital mortality in patients with AAD might have been confounded by other unknown or unmeasured parameters. Secondly, given that serum TC levels were obtained on admission, these blood samples might not have been fasting in many cases. Thirdly, only a single value of serum TC on admission was used, which might lead to exposure misclassification due to within-person variability and inability to investigate the impact of changes in serum TC level on in-hospital mortality.

In summary, our findings suggest that low TC on admission was a strong predictor of in-hospital mortality in Type-A AAD patients, but not in Type-B AAD patients. Future studies are needed to confirm these findings and to better characterize the clinical role of serum TC in patients with Type-A AAD.
